# Non-Invasive Assessment of Micro- and Macrovascular Function after Initiation of JAK Inhibitors in Patients with Rheumatoid Arthritis

**DOI:** 10.3390/diagnostics14080834

**Published:** 2024-04-17

**Authors:** Panagiota Anyfanti, Elena Angeloudi, Athanasia Dara, Eleni Pagkopoulou, Georgia-Savina Moysidou, Kleopatra Deuteraiou, Maria Boutel, Eleni Bekiari, Michael Doumas, George D. Kitas, Theodoros Dimitroulas

**Affiliations:** 1 Second Medical Department, Hippokration Hospital, Aristotle University of Thessaloniki, 54124 Thessaloniki, Greece; panyfanti@auth.gr (P.A.); elena-angeloudi@hotmail.com (E.A.); bekiarie@auth.gr (E.B.); 2 Fourth Department of Internal Medicine, Hippokration Hospital, Aristotle University of Thessaloniki, 54124 Thessaloniki, Greece; dara.athanasia@yahoo.gr (A.D.); elenipag4684@gmail.com (E.P.); deutereou@hotmail.com (K.D.); mmpoutelag@gmail.com (M.B.); 3 Rheumatology-Clinical Immunology Unit, 4th Department of Internal Medicine, Attikon University Hospital, National and Kapodistrian University of Athens Medical School, 11527 Athens, Greece; georgiamiems@gmail.com; 4 2nd Propedeutic Department of Internal Medicine, Hippokration Hospital, Aristotle University of Thessaloniki, 54124 Thessaloniki, Greece; michalisdoumas@yahoo.co.uk; 5 Department of Rheumatology, Russells Hall Hospital, Dudley Group NHS Foundation Trust, Dudley DY1 2HQ, UK; george.kitas@nhs.net; 6 School of Sport, Exercise and Rehabilitation Sciences, University of Birmingham, Birmingham B15 2TT, UK

**Keywords:** rheumatoid arthritis, Janus kinase inhibitors, arterial stiffness, carotid atherosclerosis, nailfold videocapillaroscopy

## Abstract

Background: Janus kinase (JAK) inhibitors constitute a novel class of oral biologic disease-modifying antirheumatic drugs for patients with rheumatoid arthritis (RA). However, their use has been associated with increased risk of major cardiovascular events. We investigated whether treatment with JAK inhibitors exerts significant alterations in the micro- and microvasculature in RA patients. Methods: Thirteen patients with RA initiating treatment with JAK inhibitors were prospectively studied. Eventually, data from 11 patients who completed the study were analyzed. Procedures were performed at baseline and 3 months after treatment. Nailfold videocapillaroscopy was applied to detect alterations of the dermal capillary network. Participants underwent 24 h ambulatory blood pressure monitoring (Mobil-O-Graph device) for the assessment of blood pressure (both brachial and aortic) and markers of large artery stiffening [pulse wave velocity (PWV), augmentation index] throughout the whole 24 h and the respective day- and nighttime periods. Carotid intima–media thickness was assessed with ultrasound. Results: Three-month treatment with JAK inhibitors was not associated with any differences in brachial and aortic blood pressure, arterial stiffness, and carotid atherosclerosis, with the only exception of nighttime PWV, which was significantly elevated at follow-up. However, three-month treatment with JAK inhibitors induced significant microvascular alterations and increased the total number of capillaroscopic abnormalities. Conclusions: Three-month treatment with JAK inhibitors may exert significant effects on microcirculation as assessed with nailfold videocapillaroscopy, whereas macrovascular structure and function appears largely unaffected. Further research toward this direction may add substantial information to the available literature regarding cardiovascular aspects of JAK inhibitors in RA.

## 1. Introduction

RA is an immune-mediated systemic disease characterized by synovial inflammation and hypertrophy. The activation of T-cells represents the initial event culminating in the secretion of soluble cytokines, which interact with several immune cells: namely, B-cells, macrophage and fibroblast-like synovial cells, all of which aberrantly produce inflammatory cytokines such as tumor necrosis factor-a, interleukin-1, intrleukin-6 and other mediators of inflammation [1]. Such cytokines further enhance the proliferation and differentiation of activated cells, leading to the propagation of both local and systemic inflammation accompanied by joint damage, debilitating chronic pain, and impaired functioning as well as vascular and endothelial injury. In that respect, the primary objective of RA treatment with conventional, biologic and synthetic targeted disease modifying drugs targeting the attenuation of inflammatory process is to preserve joint function and reduce the long-term effects of systemic inflammation. Modern treatment strategies suggest the introduction of anti-cytokine therapy: for example, tumor necrosis factor or interleukin-6 inhibitors in RA individuals without adequate response to conventional disease-modifying drugs have shown beneficial effects in suppressing systemic inflammation and restoring functional status [2]. JAK inhibitors are a new class of targeted therapies which have demonstrated very good efficacy in achieving low disease activity and/or remission in RA [3,4]. While multiple extra-articular manifestations and comorbidities are associated with RA, the increased cardiovascular burden is considered inherent to the disease and represents a leading cause of death in RA, as it is amplified by as much as 50% compared to the general population [5].

A large body of evidence indicates that microvascular endothelial dysfunction is the earliest precursor in the progression of cardiovascular disease (CVD), as it precedes and predicts the development of conduit artery atherosclerosis and related clinical manifestations [6]. At the same time, the increased prevalence of traditional CVD risk factors such as hypertension further drives CVD risk in RA patients. Overall, a complex interplay between chronic inflammation, the accumulation of traditional cardiovascular risk factors and the effects of antirheumatic treatment modalities is considered to form the pathophysiological basis of vascular dysfunction, atherosclerosis and related cardiovascular complications in RA [7]. With regard to pharmaceutical treatment, the vascular effects of cornerstone RA treatments such as corticosteroids or disease-modifying antirheumatic drugs (DMARDS) have been extensively studied. In contrast, further investigation is needed regarding the CVD profile of newly emerging therapeutic categories approved for the management of RA, such as Janus kinase inhibitors (JAKs) [8].

JAK inhibitors are small-molecule drugs with good long-term efficacy for RA treatment. Classified as biological DMARDs, they uniquely offer patients an oral route of administration through their ability to pass the lipid bilayer of the cellular membrane offering [9]. However, major safety concerns have been raised regarding the increased risk of CVD and thromboembolic events associated with their use [10]. Remarkably, no apparent mechanism of action has been proven to mediate this safety signal [11]. It could be hypothesized that JAK inhibition adversely affects CVD risk factors such as blood pressure, or that it exerts direct effects on the micro- microvasculature, eventually leading to the establishment of overt CVD complications.

Hence, the present study aimed to investigate whether treatment with JAK inhibitors is associated with functional and structural alterations of the micro- and macrovasculature in patients with RA. To this end, a thorough assessment of the dermal capillary network and several well-established markers of macrovascular morphology and function (arterial stiffness, carotid atherosclerosis) was performed at baseline and within 3 months’ time following the initiation of JAK inhibitors.

## 2. Methods

The study design and setting have been described in detail elsewhere [12]. Briefly, this prospective observational study enrolled adult patients with RA who were eligible for treatment with JAK inhibitors (tofacitinib, baricitinib or upadacitinib) for the first time based on international recommendations (European League Against Rheumatism and America College of Rheumatology, EULAR 2020) [13]. Exclusion criteria included the inability to understand and sign the informed consent, previous exposure to JAK inhibitors, concomitant active malignancy or any other disease with poor prognosis, recent CVD event (myocardial infarction, unstable angina, stroke) within the past 6 months, and stage III–IV heart failure according to the New York Heart Association (NYHA) criteria [14]. The immunosuppressive drugs were prescribed as per indication and relevant approved dose (for example, 15 mg daily for upatacitinib, 5 mg bd for tofacitinib and 5 mg daily for baricitinib). Before the initiation of JAK inhibitors, all patients underwent a baseline assessment of medical history and current medical treatment, anthropometric measurements, blood pressure recording, clinical examination and assessment of disease severity with calculation of DAS28 (Disease Activity Score in 28 joints). All procedures and vascular assessments were performed before the initiation of JAK inhibitors and were repeated 3 months following the baseline visit, whilst the patients were under treatment with JAK inhibitors. The study was approved from the Aristotle University Ethics Committee and the Hippokration General Hospital Scientific Board Committee, and all participants provided written informed consent before inclusion in the study.

### 2.1. Study Procedures

- Nailfold videocapillaroscopy

Nailfold videocapillaroscopy was applied to non-invasively assess in vivo morphological abnormalities or disorder of the capillary architecture of the dermal microvascular network (Optilia Digital Capillaroscope, Optilia Instruments AB, Sollentuna, Sweden). A standardized protocol was applied with the patient seated at room temperature (22–23 °C), as has been described in detail elsewhere [15,16]. Pictures were captured from the 2nd to the 5th finger of each hand using the 200× magnification video camera, and they were further analyzed with Optipix capillaroscopy software 1.7.x. The following parameters were qualitatively and/or quantitatively evaluated: capillary density, defined as the number of capillaries in the first row in 1 mm (capillaries/mm), capillary dimensions (width and length), microhemorrhages, avascular areas, and the presence of ramified, bushy, crossed and tortuous capillaries. These parameters were determined on each nail, and the average of all measurements for each patient were finally calculated.

- Measurement of blood pressure, arterial stiffness and hemodynamic parameters

Current hypertension guidelines recommend 24 h ambulatory blood pressure recording as the ideal method of BP monitoring [17]. All participants underwent measurements with the Mobil-O-Graph (IEM, Stolberg, Germany), which enables the concomitant automatic calculation of arterial stiffness and hemodynamic parameters at each blood pressure measurement through central aortic waveform analysis. This device has been approved by Food and Drug Administration (FDA) and the European Union and has been validated according to the protocols of the European and the British Society of Hypertension [18,19]. A minimum of 70% successful readings was regarded as technically sufficient for each recording. Blood pressure was automatically measured at 20-min intervals during the day and at 30-min intervals during the night to obtain mean 24 h, day- and nighttime blood pressure. Moreover, mean values of arterial stiffness [pulse wave velocity (PWV), augmentation index (AIx)] and hemodynamic parameters [central (aortic) blood pressure] were obtained throughout the whole 24 h, day- and nighttime period and used in the analysis.

- Carotid atherosclerosis

Carotid intima–media thickness (cIMT) was measured as a well-acknowledged index of carotid atherosclerosis and a surrogate measure of atherosclerotic CVD. cIMT was calculated from longitudinal images obtained with ultrasound (Aloka Pro Sound A7, Ultrasound System, Tokyo, Japan) in the far wall of the distal 10 mm of each artery. Briefly, cIMT measurement was performed in a region free of plaque where there is a standard double-line pattern. The sections of the arterial walls were evaluated in a longitudinal view, strictly perpendicular to the ultrasound beam, with both walls being visible to achieve diameter measurements, and cIMT was measured on the proximal vessel wall.

- Blood tests

Routine blood tests were performed for the evaluation of hematological and biochemical parameters (glucose, lipids, renal function, uric acid) and inflammatory markers [erythrocyte sedimentation rate (ESR), C-reactive protein (CRP)].

### 2.2. Statistical Analysis

Statistical analysis was performed using the Statistical Package for Social Sciences, SPSS Inc., Chicago, IL, USA software, version 22. Descriptive statistical tests were used to present the cohort main characteristics. The Shapiro–Wilk normality test was used to evaluate the distribution of quantitative variables. Normally distributed variables were expressed as mean ± standard deviation (SD), while non-normal variables were described as median values (interquartile range). Categorical variables were presented as frequencies and percentages. Differences in outcomes from baseline to three months after treatment were evaluated with the parametric paired-samples *t*-test or the non-parametric Wilcoxon signed-rank test, according to the normality of the data distribution. A probability value of *p* ≤ 0.05 was considered statistically significant.

## 3. Results

Thirteen patients with RA treated with JAK inhibitors as per the treating physician decision were enrolled. Eventually, two patients dropped out before the end of the study, and 11 patients who completed the study were included in data analysis. Characteristics of the 11 patients who were eligible for further data analysis are presented in Table 1. Of them, five patients were prescribed upadacitinib, four patients were prescribed baricitinib and two patients were prescribed tofacitinib at the beginning of the study, as per the treating physician’s decision. Of the three patients previously under biologics, two had been receiving etanercept and one had been receiving adalimumab before switching to JAK inhibitors. A washout of two weeks was applied for the three patients under etanercept or adalimumab, which is expected to cover the half-life of both drugs. We confirm that the baseline characteristics of the two dropouts did not significantly differ from those of the 11 included patients (data not shown).

- *Effects of JAK inhibitors on hematological parameters*

As presented in Table 2, routine hematological parameters, inflammatory markers, metabolic profile and renal function did not change from baseline with the only exception of triglycerides, which significantly decreased three months following treatment.

- *Effects of JAK inhibitors on peripheral and central hemodynamics*

Office blood pressure did not change during treatment with JAK inhibitors, and the same was observed for both peripheral (brachial) and central (aortic) blood pressure assessed throughout the whole 24 h period and the respective day- and nighttime intervals (Table 3 (a,b)).

- *Effects of JAK inhibitors on macrovascular parameters*

Changes in measures of macrovascular morphology and function are presented in Table 3. Both measures of arterial stiffness (PWV and AIx) remained unchanged over treatment, as assessed throughout the whole 24 h period and the respective day- and nighttime windows. The only exception was nighttime PWV, which significantly increased after three months (Table 3 (c)). Likewise, there was no difference in cIMT between 3 months and baseline (Table 3 (d)).

- Effects of JAK inhibitors on microvascular capillaroscopic parameters

Contrary to markers of macrovascular pathology, several capillaroscopic parameters were affected from therapy with JAK inhibitors. More specifically, treatment with JAK inhibitors significantly decreased venous limb, apical width and capillary length, and increased the number of ramified capillaries and the total number of abnormalities. Changes in microvascular capillaroscopic parameters from baseline to three months are presented in detail in Table 4.

## 4. Discussion

The present study suggests that treatment with JAK inhibitors may induce significant alterations in microvascular capillaroscopic parameters within three months’ time. By contrast, markers of macrovascular function and morphology, i.e., arterial stiffness and carotid atherosclerosis, remain largely unaffected. Findings from the present study imply that subclinical microvascular injury might mediate the adverse cardiovascular effects of JAK inhibitors in patients with RA.

The early initiation of effective therapy in RA alleviates the inflammatory burden and at the same time prevents further vascular damage and the emergence of adverse CVD events [20]. The rationale for the use of JAK inhibitors in RA is supported by a solid pathophysiological background. Inhibition of the JAK–STAT pathway is responsible for many pathophysiological alterations due to the blockade of several cytokines and interferons (IFNs). More specifically, JAK1 is responsible for the transmission of signals by IL-6, IL-10, IL-11, IL-19, IL-20, and IL-22, and IFN-α, IFN-β, and IFN-γ, while JAK2 activation is responsible for the signaling of hormone-like cytokines erythropoietin, thrombopoietin, growth hormone, GM-CSF, IL-3 and IL-5 [21]. JAK3 is primarily expressed on hematopoietic cells and transmits signals from IL-2, IL-4, IL-7, IL-9, IL-15 and IL-21 [21]. Lastly, TYK2 facilitates signaling for IL-12, IL-23 and type I IFNs [22]. Of note, experimental and preclinical studies suggest that the beneficial effects of JAK inhibitors may be largely associated with the IL-23/-17 axis, which plays a dominant role in the underlying pathogenesis of autoimmune rheumatic disorders [6,23]. Although IL-17 does not seem directly modulated by the JAK/STAT system [24], the function of IL-23, which is an upstream driver of IL-17A release, is regulated by the JAK2–TYK2/STAT3–STAT4 system [25,26].

Despite their clinical efficacy, major concerns regarding cardiovascular aspects of JAK inhibitors have been raised recently. These mainly emerge from the post hoc analysis of the ORAL surveillance, which is a randomized, open-label, noninferiority, postauthorization, safety end-point trial. The study revealed that RA patients with CVD risk factors receiving tofacitinib had a greater likelihood of experiencing CVD events compared to patients treated with tumor necrosis factor (TNF) inhibitors [10]. Potential underlying mechanisms remain unknown but are presumably related to the pleiotropic downstream effects of the JAK–STAT (signal transducer and activator of transcription proteins) signaling pathway, which may not always favor cardiovascular function. However, potential mechanisms underlying cardiovascular impairment in patients treated with JAK inhibitors emerge almost exclusively from experimental data [21].

In contrast, a very limited number of studies have investigated in vivo potential cardiotoxic or vasculodestructive processes triggered by JAK inhibition in patients with RA. Vascular effects of biologic and targeted synthetic antirheumatic drugs approved for RA have been recently reviewed. The study concluded that although many studies have shown a beneficial effect of biologics on vascular function and endothelial injury, the overall impact of JAKi and rituximab remains inconclusive [27]. More specifically, a study by Kerekes et al. investigated the effects of tofacitinib therapy in relation to vascular function and inflammation on 30 RA patients receiving a dose of 5 mg or 10 mg twice daily [28]. Following one year of therapy, patients had decreased IL-6, VEGF, bFGF, EGF, PIGF, CathK and increased Gal-3 production. Furthermore, RF-seropositive patients had higher bFGF, PlGF and NT-proBNP levels. Overall, study findings indicated that tofacitinib therapy inhibits synovial and aortic inflammation by decreasing bFGF, PlGF and IL-6 production. The effects of tofacitinib therapy were assessed in 30 RA patients in relation to inflammation, functional and pathological vascular changes [29]. Serum L-arginine, L-citrulline, L-ornithine, inducible nitric oxide synthase (iNOS), asymmetric (ADMA) and symmetric dimethylarginine (SDMA), L-N-monomethyl-arginine (L-NMMA), cysteine, homocysteine, and methionine concentrations were assessed at baseline as well as at 6 and at 12 months of treatment. Vascular function was evaluated using brachial artery flow-mediated vasodilation (FMD), carotid intima–media thickness (IMT) and pulse-wave velocity (PWV) by ultrasound. Findings suggested that tofacitinib treatment improved functional status by promoting arginine and methionine production and by suppressing systemic inflammation.

Findings from the present study extend the above-mentioned data, as a number of capillaroscopic parameters were affected. More specifically, nailfold videocapillaroscopy revealed decreased venous capillary limb, apical width and capillary length as well as higher frequency of ramified capillaries and total abnormalities. Nailfold videocapillaroscopy is a useful, non-invasive method to assess the microvasculature in real time and in vivo, by identifying qualitative and quantitative capillaroscopic changes [30]. Various capillaroscopic parameters may be affected in patients with RA [31], and their clinical significance has been recently reviewed [32]. Definite evidence from specifically designed prospective studies providing a direct link between alterations detected with nailfold videocapillaroscopy and future adverse cardiovascular events is currently missing. However, the presence of such alterations has been consistently associated with generalized vascular impairment [15,16]. Based on the results from the present study, it could be hypothesized that treatment with JAK inhibitors in patients with RA induces relatively short-term morphological alterations of the microvasculature, which in the long term may affect microvascular function and promote large vessel impairment through a cross-talk between the micro- and microvasculature [33]. Still, the study findings need to be interpreted with caution and regarded as hypothesis generating in the light of the small study sample. It needs to be underlined that the clinical significance of the observed capillaroscopic alterations (decrease in venous capillary limb, apical width and capillary length, and increase in capillary ramification) is not fully understood. Whether these changes might be accompanied by ultimately adverse, neutral, or even beneficial effects in terms of cardiovascular health cannot be deduced from the present study.

On the other hand, noteworthy changes in several well-established parameters of macrovascular function and morphology were not observed after three months of treatment, including brachial and aortic blood pressure, arterial stiffness and carotid atherosclerosis. However, it needs to be taken into account that the follow-up time might be too short to allow for significant changes in PWV and cIMT to be detected. This is particularly true for cIMT, whose alterations may be documented after several months or even years. Indeed, a previous study aiming to estimate atherosclerosis progression in patients with RA showed that after a mean of 2.8 years, the cIMT increased by 0.050 mm, *p* ≤ 0.001, which corresponds to a progression rate of 0.018 mm/year [34]. On the other hand, changes in PWV may occur sooner than changes in cIMT, but these may still need several weeks or months to be detected. Wong et al. assessed the effects of infliximab treatment on arterial stiffness and were able to detect changes in PWV more than a year later (at 56 weeks) yet not at 6 months. Remarkably, cIMT did not change at all over the study period [35]. Based on the above, it may be hard to expect any changes in either PWV or cIMT within the three-month follow-up period of our study, and longer follow-up studies or with a control arm receiving conventional treatment are warranted.

Strengths of the present study include a solid methodological approach that enabled the thorough assessment of both the micro- and the macrovasculature. Reliable, well-established markers of large artery stiffening and atherosclerosis were used, and a standardized nailfold videocapillaroscopy protocol was applied that has been verified in several previous studies. To our best knowledge, this is the first time that the effects of JAK targeting on the above-mentioned micro- and macrovascular markers have been analyzed in the same study.

However, the study is limited by the small study population, and results may be regarded as preliminary pending confirmation from further studies. Importantly, a comparison group was not included. Although this was beyond the scope of our study, the inclusion of a control group would have added value to the study findings, as any observed differences in outcomes could be more safely attributed to treatment with JAK inhibitors. Hence, although interesting, these preliminary data have to be confirmed in future studies using a control group. Three patients were previously prescribed biologics which have been associated with improvements in arterial stiffness and carotid atherosclerosis [36,37], but a two-week washout was applied before inclusion in the study. Capillaroscopic images were reviewed by a single investigator, although at least two independent investigators might be used to ensure inter-observer repeatability. Nevertheless, the protocol of the study has been previously validated in several other studies from the study group. Still, non-invasive assessment of the micro- and macrovascular is a promising approach in RA, but it requires pre-specified methodology for the evaluation of certain parameters and the application of a homogeneous scoring system to ensure the validity of measurements as the international community (EULAR) did for systemic sclerosis [38]. Last but not least, follow-up was limited to three months, and no conclusions can be made regarding the long-term vascular effects of these drugs.

## 5. Conclusions

In conclusion, three-month treatment with JAK inhibitors in RA patients resulted in altered morphological features of the dermal capillary network as detected non-invasively with nailfold videocapillaroscopy. This finding implies that treatment with JAK inhibitors in patients with RA induces relatively short-term morphological alterations of the microvasculature, which may subsequently promote altered vascular function. By contrast, JAK inhibition was not associated with macrovascular impairment, as shown by largely unaffected markers of large artery stiffening and carotid atherosclerosis. Although the present study findings may be considered preliminary, these results suggest that JAK inhibition may trigger microvascular dysfunction. Further prospective studies are needed to clarify whether such microvascular alterations might contribute, at least partially, to subsequent cardiovascular complications related to treatment with JAK inhibitors in RA.

## Figures and Tables

**Table 1:** Patient characteristics.

	JAK Inhibitors-Treated Patients (*n* = 11)
Age (years)	55.0 ± 6.3
Female/male ratio	9:2
Disease duration (months)	160.4 ± 88.2
BMI (kg/m^2^)	25.7 ± 4.4
Current smoking, *n* %	7 (63.6)
Hypertension, *n* (%)	3 (27.3)
Dyslipidemia, *n* (%)	3 (27.3)
DAS28	3.8 ± 0.7
RF positivity, *n* (%)	3 (27.3)
Anti-CCP positivity, *n* (%)	8 (72.7)
Antirheumatic medication	
Methotrexate, *n* (%)	4 (36.4)
Corticosteroids, *n* (%)	5 (45.5)
Biologics, *n* (%)	3 (27.3)
Cardiovascular drugs	
RAAS inhibitors, *n* (%)	3 (27.3)
Calcium channel blockers, *n* (%)	1 (9.1)
Beta blockers, *n* (%)	1 (9.1)
Diuretics, *n* (%)	2 (18.2)
Statins, *n* (%)	3 (27.3)

JAK: Janus kinase; BMI: body mass index; DAS28: disease activity score in 28 joints; RF: rheumatoid factor; anti-CCP: anti-cyclic citrullinated peptides; RAAS: renin–angiotensin–aldosterone system. Continuous variables are presented as mean ± SD according to normality tests.

**Table 2:** Changes in routine and disease-related laboratory measurements from baseline following three-month treatment with JAK inhibitors.

	JAK Inhibitors-Treated Patients		
	Baseline	Follow-Up	*p* Value
**ESR (mm/h)**	22.5 ± 8.4	18.0 ± 10.4	0.316
**CRP (mg/L)**	2.0 (6.5)	2.0 (2.7)	1.000
**WBC 10^3^/μL**	7.9 ± 2.7	7.1 ± 2.3	0.311
**Hematocrit (%)**	38.8 ± 3.0	38.2 ± 2.2	0.527
**Hemoglobin (g/dL)**	12.9 ± 1.1	12.8 ± 0.7	0.612
**Platelets (10^3^/μL)**	278.4 ± 53.9	278.7 ± 49.5	0.973
**Urea (mg/dL)**	36.1 ± 12.3	37.9 ± 11.7	0.497
**Creatinine (mg/dL)**	0.77 ± 0.16	0.81 ± 0.11	0.109
**Uric acid (mg/dL)**	4.6 ± 1.4	4.9 ± 1.2	0.365
**Glucose (mg/dL)**	87 (14)	92 (17)	0.283
**HDL-C (mg/dL)**	59.4 ± 16.6	62.9 ± 20.5	0.318
**LDL (mg/dL)**	119.9 ± 30.8	126.0 ± 34.7	0.405
**Total cholesterol (mg/dL)**	195 (13)	195 (58)	0.859
**Triglycerides (mg/dL)**	125.8 ± 46.6	98.0 ± 32.7	0.020

JAK: Janus kinase; ESR: erythrocyte sedimentation rate; CRP: C-reactive protein; WBC: white blood cells; HDL-C: high-density lipoprotein cholesterol; LDL-C: low-density lipoprotein cholesterol; Continuous variables are presented as mean ± SD or median (interquartile range) according to normality tests.

**Table 3:** Changes in (a) blood pressure measurements, (b) central hemodynamics, (c) arterial stiffness and (d) carotid atherosclerosis, from baseline following three-month treatment with JAK inhibitors.

	JAK Inhibitors-Treated Patients		
	Baseline	Follow-Up	*p* Value
***(a) Brachial blood pressure measurements***			
Office SBP (mmHg)	132.6 ± 20.7	133.0 ± 18.2	0.931
Office DBP (mmHg)	80.4 ± 9.8	80.7 ± 8.4	0.899
24 h SBP (mmHg)	116.9 ± 14.7	117.5 ± 14.7	0.860
24 h DBP (mmHg)	75.8 ± 11.3	73.6 ± 7.8	0.524
Daytime SBP (mmHg)	119.2 ± 14.9	120.4 ± 6.7	0.711
Daytime DBP (mmHg)	78.2 ± 12.2	76.6 ± 8.3	0.655
Nighttime SBP (mmHg)	110.3 ± 14.7	111.0 ± 9.6	0.854
Nighttime DBP (mmHg)	69.0 ± 9.7	66.6 ± 7.6	0.531
***(b) Central hemodynamics***			
24 h aortic SBP (mmHg)	117.9 ± 14.8	120.4 ± 8.9	0.554
24 h aortic DBP (mmHg)	76.4 ± 11.1	74.4 ± 8.1	0.596
Daytime aortic SBP (mmHg)	118.0 ± 14.1	121.6 ± 8.5	0.321
Daytime aortic DBP (mmHg)	78.6 ± 11.8	77.6 ± 8.8	0.793
Nighttime aortic SBP (mmHg)	110.3 ± 14.7	111.0 ± 9.6	0.685
Nighttime aortic DBP (mmHg)	69.0 ± 9.7	66.6 ± 7.6	0.473
***(c) Arterial stiffness***			
24 h PWV (m/s)	7.4 ± 0.7	7.7 ± 1.0	0.086
Daytime PWV (m/s)	7.5 ± 0.7	7.8 ± 1.0	0.105
Nighttime PWV (m/s)	7.2 ± 0.7	7.6 ± 1.0	**0.017**
24 h AIx (%)	24.5 ± 6.6	26.9 ± 6.7	0.223
Daytime AIx (%)	25.1 ± 6.3	26.6 ± 6.1	0.451
Nighttime AIx (%)	22.5 ± 8.8	28.3 ± 10.2	0.059
***(d) Carotid atherosclerosis***			
IMT, right carotid artery (mm)	0.60 ± 0.11	0.61 ± 0.11	0.801
IMT, left carotid artery (mm)	0.64 ± 0.19	0.69 ± 0.17	0.301
Mean carotid IMT (mm)	0.62 ± 0.15	0.65 ± 0.14	0.471

JAK: Janus kinase; SBP: systolic blood pressure; DBP: diastolic blood pressure; PWV: pulse wave velocity; AIX: augmentation index; IMT: intima–media thickness; continuous variables are presented as mean ± SD according to normality tests.

**Table 4:** Changes in capillaroscopic parameters from baseline following three-month treatment with JAK inhibitors.

	Baseline	Follow-Up	*p* Value
Capillary density (*n*/mm^2^)	9.0 (2.0)	9.5 (1.0)	0.763
Avascular areas, *n* (%)	3 (27.3)	6 (54.5)	0.375
Capillary width (μm)	31.0 (9.5)	33.3 (5.4)	0.214
Arterial limb (μm)	10.6 ± 2.1	10.7 ± 1.3	0.970
Venous limb (μm)	12.5 ± 2.1	11.4 ± 1.6	0.047
Apical width (μm)	28.5 ± 4.7	25.5 ± 2.9	0.044
Capillary length (μm)	164.8 ± 53.8	133.4 ± 41.5	0.028
Internal diameter	13.2 ± 2.2	13.3 ± 2.0	0.849
Microhemorrhages, *n* (%)	2 (18.2)	0 (0)	0.500
Ramified capillaries (loops/mm^2^)	0.5 (1.0)	1 (1)	0.020
Bushy capillaries (loops/mm^2^)	0 (0)	0 (0)	0.564
Tortuous capillaries (loops/mm^2^)	1 (1)	2 (1)	0.214
Crossed capillaries (*n*/mm^2^)	2 (1)	2 (2)	0.157
Subpapillary venous plexus, *n* (%)	5 (45.5)	5 (45.5)	1.000
Total number of abnormalities, *n*	3.6 ± 1.6	5.2 ± 1.2	0.036

Continuous variables are presented as mean ± SD or median (interquartile range) according to normality tests.

## Data Availability

The data presented in this study are available on request from the corresponding author.

## References

[1] Jahid M., Khan K.U., Rehan-Ul-Haq, Ahmed R.S. (2023). Overview of Rheumatoid Arthritis and Scientific Understanding of the Disease. Mediterr. J. Rheumatol..

[2] Smolen J.S., Landewé R.B.M., Bergstra S.A., Kerschbaumer A., Sepriano A., Aletaha D., Caporali R., Edwards C.J., Hyrich K.L., Pope J.E. (2023). EULAR Recommendations for the Management of Rheumatoid Arthritis with Synthetic and Biological Disease-Modifying Antirheumatic Drugs: 2022 Update. Ann. Rheum. Dis..

[3] Szekanecz Z., Buch M.H., Charles-Schoeman C., Galloway J., Karpouzas G.A., Kristensen L.E., Ytterberg S.R., Hamar A., Fleischmann R. (2024). Efficacy and Safety of JAK Inhibitors in Rheumatoid Arthritis: Update for the Practising Clinician. Nat. Rev. Rheumatol..

[4] Weng C., Xue L., Wang Q., Lu W., Xu J., Liu Z. (2021). Comparative Efficacy and Safety of Janus Kinase Inhibitors and Biological Disease-Modifying Antirheumatic Drugs in Rheumatoid Arthritis: A Systematic Review and Network Meta-Analysis. Ther. Adv. Musculoskelet. Dis..

[5] Aviña-Zubieta J.A., Choi H.K., Sadatsafavi M., Etminan M., Esdaile J.M., Lacaille D. (2008). Risk of Cardiovascular Mortality in Patients with Rheumatoid Arthritis: A Meta-Analysis of Observational Studies. Arthritis Care Res..

[6] Bordy R., Totoson P., Prati C., Marie C., Wendling D., Demougeot C. (2018). Microvascular Endothelial Dysfunction in Rheumatoid Arthritis. Nat. Rev. Rheumatol..

[7] Semb A.G., Ikdahl E., Wibetoe G., Crowson C., Rollefstad S. (2020). Atherosclerotic Cardiovascular Disease Prevention in Rheumatoid Arthritis. Nat. Rev. Rheumatol..

[8] Chatzidionysiou K. (2020). Beyond Methotrexate and Biologics in RA—Efficacy of JAK Inhibitors and Their Place in the Current Treatment Armamentarium. Mediterr. J. Rheumatol..

[9] Harrington R., Al Nokhatha S.A., Conway R. (2020). JAK Inhibitors in Rheumatoid Arthritis: An Evidence-Based Review on the Emerging Clinical Data. J. Inflamm. Res..

[10] Winthrop K.L., Cohen S.B. (2022). Oral Surveillance and JAK Inhibitor Safety: The Theory of Relativity. Nat. Rev. Rheumatol..

[11] Singh J.A. (2022). Risks and Benefits of Janus Kinase Inhibitors in Rheumatoid Arthritis—Past, Present, and Future. N. Engl. J. Med..

[12] Anyfanti P., Angeloudi E., Pagkopoulou E., Bekiari E. (2022). JAK Inhibition in Patients with Rheumatoid Arthritis: Haemodynamic Effects and Impact on Micro- and Macrovascular Function. Study Design and Rationale. Mediterr. J. Rheumatol..

[13] Smolen J.S., Landewé R.B.M., Bijlsma J.W.J., Burmester G.R., Dougados M., Kerschbaumer A., McInnes I.B., Sepriano A., van Vollenhoven R.F., de Wit M. (2020). EULAR Recommendations for the Management of Rheumatoid Arthritis with Synthetic and Biological Disease-Modifying Antirheumatic Drugs: 2019 Update. Ann. Rheum. Dis..

[14] Dolgin M., New York Heart Association Criteria Committee (1994). Nomenclature and Criteria for Diagnosis of Diseases of the Heart and Great Vessels Nomenclature and Criteria for Diagnosis of Diseases of the Heart and Great Vessels.

[15] Angeloudi E., Anyfanti P., Dara A., Pagkopoulou E., Bekiari E., Sgouropoulou V., Garyfallos A., Doumas M., Kitas G.D., Dimitroulas T. (2023). Peripheral Nailfold Capillary Microscopic Abnormalities in Rheumatoid Arthritis Are Associated with Arterial Stiffness: Results from a Cross-Sectional Study. Microvasc. Res..

[16] Minopoulou I., Theodorakopoulou M., Boutou A., Arvanitaki A., Pitsiou G., Doumas M., Sarafidis P., Dimitroulas T. (2021). Nailfold Capillaroscopy in Systemic Sclerosis Patients with and without Pulmonary Arterial Hypertension: A Systematic Review and Meta-Analysis. J. Clin. Med..

[17] Mancia G., Kreutz R., Brunström M., Burnier M., Grassi G., Januszewicz A., Muiesan M.L., Tsioufis K., Agabiti-Rosei E., Algharably E.A.E. (2023). 2023 ESH Guidelines for the Management of Arterial Hypertension The Task Force for the Management of Arterial Hypertension of the European Society of Hypertension. J. Hypertens..

[18] Wei W., Tölle M., Zidek W., van der Giet M. (2010). Validation of the Mobil-O-Graph: 24 h-Blood Pressure Measurement Device. Blood Press. Monit..

[19] Franssen P.M.L., Imholz B.P.M. (2010). Evaluation of the Mobil-O-Graph New Generation ABPM Device Using the ESH Criteria. Blood Press. Monit..

[20] Aletaha D., Smolen J.S. (2002). The Rheumatoid Arthritis Patient in the Clinic: Comparing More than 1300 Consecutive DMARD Courses. Rheumatology.

[21] Reddy V., Cohen S. (2020). JAK Inhibitors: What Is New?. Curr. Rheumatol. Rep..

[22] Tokumasa N., Suto A., Kagami S., Furuta S., Hirose K., Watanabe N., Saito Y., Shimoda K., Iwamoto I., Nakajima H. (2007). Expression of Tyk2 in Dendritic Cells Is Required for IL-12, IL-23, and IFN-γ Production and the Induction of Th1 Cell Differentiation. Blood.

[23] Schinocca C., Rizzo C., Fasano S., Grasso G., La Barbera L., Ciccia F., Guggino G. (2021). Role of the IL-23/IL-17 Pathway in Rheumatic Diseases: An Overview. Front. Immunol..

[24] Gaffen S.L. (2009). Structure and Signalling in the IL-17 Receptor Family. Nat. Rev. Immunol..

[25] Banerjee S., Biehl A., Gadina M., Hasni S., Schwartz D.M. (2017). JAK–STAT Signaling as a Target for Inflammatory and Autoimmune Diseases: Current and Future Prospects. Drugs.

[26] Raychaudhuri S.K., Abria C., Raychaudhuri S.P. (2017). Regulatory Role of the JAK STAT Kinase Signalling System on the IL-23/IL-17 Cytokine Axis in Psoriatic Arthritis. Ann. Rheum. Dis..

[27] Gerganov G., Georgiev T., Dimova M., Shivacheva T. (2023). Vascular Effects of Biologic and Targeted Synthetic Antirheumatic Drugs Approved for Rheumatoid Arthritis: A Systematic Review. Clin. Rheumatol..

[28] Kerekes G., Czókolyová M., Hamar A., Pusztai A., Tajti G., Katkó M., Végh E., Pethő Z., Bodnár N., Horváth Á. (2023). Effects of 1-Year Tofacitinib Therapy on Angiogenic Biomarkers in Rheumatoid Arthritis. Rheumatology.

[29] Soós B., Hamar A., Pusztai A., Czókolyová M., Végh E., Szamosi S., Pethő Z., Gulyás K., Kerekes G., Szántó S. (2022). Effects of Tofacitinib Therapy on Arginine and Methionine Metabolites in Association with Vascular Pathophysiology in Rheumatoid Arthritis: A Metabolomic Approach. Front. Med..

[30] Roberts-Thomson P.J., Patterson K.A., Walker J.G. (2023). Clinical Utility of Nailfold Capillaroscopy. Intern. Med. J..

[31] Rajaei A., Dehghan P., Amiri A. (2017). Nailfold Capillaroscopy in 430 Patients with Rheumatoid Arthritis. Caspian J. Intern. Med..

[32] Anyfanti P., Angeloudi E., Dara A., Arvanitaki A., Bekiari E., Kitas G.D., Dimitroulas T. (2022). Nailfold Videocapillaroscopy for the Evaluation of Peripheral Microangiopathy in Rheumatoid Arthritis. Life.

[33] Laurent S., Briet M., Boutouyrie P. (2009). Large and Small Artery Cross-Talk and Recent Morbidity-Mortality Trials in Hypertension. Hypertension.

[34] del Rincón I., Polak J.F., O’Leary D.H., Battafarano D.F., Erikson J.M., Restrepo J.F., Molina E., Escalante A. (2015). Systemic Inflammation and Cardiovascular Risk Factors Predict Rapid Progression of Atherosclerosis in Rheumatoid Arthritis. Ann. Rheum. Dis..

[35] Wong M., Oakley S.P., Young L., Jiang B.Y., Wierzbicki A., Panayi G., Chowienczyk P., Kirkham B. (2009). Infliximab Improves Vascular Stiffness in Patients with Rheumatoid Arthritis. Ann. Rheum. Dis..

[36] Vlachopoulos C., Gravos A., Georgiopoulos G., Terentes-Printzios D., Ioakeimidis N., Vassilopoulos D., Stamatelopoulos K., Tousoulis D. (2018). The Effect of TNF-a Antagonists on Aortic Stiffness and Wave Reflections: A Meta-Analysis. Clin. Rheumatol..

[37] Anghel D., Sîrbu C., Hoinoiu E.-M., Petrache O.-G., Pleșa C.-F., Negru M., Ioniţă-Radu F. (2021). Influence of Anti-TNF Therapy and Homocysteine Level on Carotid Intima-media Thickness in Rheumatoid Arthritis Patients. Exp. Ther. Med..

[38] Smith V., Herrick A.L., Ingegnoli F., Damjanov N., De Angelis R., Denton C.P., Distler O., Espejo K., Foeldvari I., Frech T. (2020). Standardisation of Nailfold Capillaroscopy for the Assessment of Patients with Raynaud’s Phenomenon and Systemic Sclerosis. Autoimmun. Rev..

